# Ketamine Use for ED Sedation: Pharmacologic Basis, Clinical Applications, and Safety Considerations

**DOI:** 10.7759/cureus.103774

**Published:** 2026-02-17

**Authors:** Sabrina Montoya, Christian Andrés Soto Cordero, Elia L Zamora, Ronald Chavarría, Diego Alvarez Ramirez

**Affiliations:** 1 Emergency Medicine, Hospital Monseñor Sanabria Martínez, Puntarenas, CRI

**Keywords:** analgesia, emergency medicine, ketamine, pharmacology, procedural sedation, safety

## Abstract

Ketamine is a versatile pharmacologic agent whose unique receptor interactions explain its broad applicability in emergency medicine. Its primary mechanism of action is non-competitive antagonism of N-methyl-D-aspartate (NMDA) receptors, which underlies its dissociative, anesthetic, and analgesic properties. By reducing excitatory neurotransmission and limiting glutamatergic activity, ketamine provides effective sedation while helping prevent neurotoxicity. Additional interactions with opioid, monoaminergic, and cholinergic systems contribute to its analgesic and psychotropic effects, reinforcing its neuroprotective and antidepressant potential. Ketamine is rapidly absorbed, crosses the blood-brain barrier efficiently, and is metabolized in the liver by cytochrome P450 enzymes CYP3A4 and CYP2B6 into active metabolites such as hydroxynorketamine. Its short half-life and multiple routes of administration, including IV, intramuscular (IM), and intranasal, allow for flexible use in different clinical contexts. In the ED, ketamine is used for procedural sedation, fracture and dislocation reduction, wound repair, and trauma management. Its dissociative properties ensure adequate analgesia and amnesia while preserving airway reflexes, reducing the need for additional anesthetic agents. It is particularly valuable in pediatric patients, providing effective sedation and pain control with minimal adverse effects. In cases of trauma, burns, or shock, the sympathomimetic activity of ketamine helps maintain cardiovascular tone and perfusion, offering an advantage over opioids, which may cause hypotension or respiratory depression. Adverse reactions are uncommon but may include laryngospasm, hypertension, tachycardia, or psychological emergence phenomena such as agitation or hallucinations. Coadministration of benzodiazepines can mitigate these effects and improve patient comfort during recovery. Standardized dosing protocols, typically 0.15-0.30 mg/kg IV for analgesia and 2-5 mg/kg IM for sedation, ensure both efficacy and safety. Comparative studies indicate that ketamine maintains hemodynamic stability better than etomidate and has a more favorable respiratory profile compared with benzodiazepine-opioid combinations. Overall, ketamine remains a cornerstone agent for emergency procedural sedation due to its rapid onset, reliability, and adaptability across a wide range of patient populations.

## Introduction and background

Ketamine has become a significant agent in ED procedural sedation due to its unique pharmacologic properties and versatility. Initially introduced in the 1970s, its use in clinical practice has progressively expanded beyond anesthetic purposes to include applications in critical care and emergency medicine. Over the decades, ketamine has proven valuable for various indications, including procedural sedation, rapid sequence intubation (RSI), and the management of agitation and pain [1].

Procedural sedation plays a central role in emergency medicine, allowing clinicians to perform painful or anxiety-inducing procedures while maintaining patient comfort and cooperation. Within this context, ketamine has emerged as one of the most widely used agents because of its rapid onset and distinctive dissociative anesthetic properties. These characteristics enable effective sedation without significant compromise of respiratory function, providing an advantage over other sedatives commonly employed in the ED [1,2].

From a historical perspective, ketamine was synthesized in 1962 by Calvin Stevens at Parke-Davis as part of efforts to develop a safer alternative to phencyclidine (PCP). Initially designated CI-581, the compound was structurally derived from PCP, which was known for its dissociative properties but associated with significant adverse effects. Ketamine was subsequently approved for clinical use in 1970, and its early clinical applications marked a turning point in anesthetic practice. While initially developed as an alternative to traditional anesthetics, ketamine quickly demonstrated broader potential across medical disciplines. Over time, its use evolved from strictly anesthetic applications to encompass roles in sedation for agitation and pain management, reflecting its growing relevance in emergency and critical care settings [1,3].

The renewed focus on ketamine in emergency medicine is multifactorial. In terms of efficacy, it provides reliable sedation and analgesia, with multiple studies confirming successful outcomes at both dissociative and sub-dissociative doses [2,4]. Its safety profile further supports its widespread use, as adverse events in ED settings remain relatively uncommon. Notably, sub-dissociative doses are associated with fewer complications compared with higher doses, enhancing its suitability for a wide range of patient populations [2,3].

In addition to its safety and efficacy, ketamine’s versatility contributes to its prominence. Its capacity to manage conditions ranging from severe agitation to acute pain exemplifies its adaptability within the ED. Furthermore, combining ketamine with other sedatives, such as propofol, can optimize procedural outcomes and improve patient tolerance [1,5].

The objective of this review is to examine and synthesize current evidence regarding the use of ketamine for procedural sedation in the ED. This includes an analysis of its pharmacologic foundations, clinical efficacy, safety profile, and versatility across diverse emergency care settings.

## Review

Methodology

This study was conducted as a narrative review aimed at providing a structured, qualitative synthesis of current evidence regarding the use of ketamine for procedural sedation in the ED. It was not designed as a systematic review and therefore did not follow PRISMA guidelines, nor did it include a formal risk-of-bias assessment, hierarchical grading of study designs, structured data extraction tables, or quantitative statistical synthesis. The objective was to integrate and critically discuss recent literature rather than perform a reproducible meta-analytic evaluation.

The literature search focused primarily on studies published between January 2020 and December 2025 to prioritize the most current evidence in emergency medicine practice. Searches were conducted in PubMed, Scopus, and Web of Science using combinations of the following terms: “ketamine”, “procedural sedation”, “emergency medicine”, “analgesia”, “pharmacology”, and “safety”. Articles were eligible if published in English or Spanish and if they addressed pharmacological mechanisms, clinical indications, dosing strategies, safety outcomes, or comparisons with other sedative agents. Although the primary time frame began in 2020, selected landmark pre-2020 publications were included when necessary to provide historical context or clarify evolving standards, particularly regarding pediatric sedation and intracranial pressure considerations.

A total of 29 sources were included, comprising original research articles, systematic reviews, clinical trials, meta-analyses, and practice guidelines from recognized emergency medicine and anesthesiology organizations. The analysis was qualitative and thematic. Evidence was grouped into categories including pharmacologic foundations, clinical applications, safety profile, dosing strategies, and comparative effectiveness. No pooled statistical estimates, heterogeneity analyses, or formal meta-analytic procedures were performed.

AI tools were used exclusively for organizational support, language refinement, and thematic structuring. All literature selection, interpretation of findings, critical analysis, and final conclusions were conducted and validated solely by the authors. This narrative and comparative approach allowed for a comprehensive and clinically oriented overview of ketamine’s current role in ED sedation while maintaining transparency regarding methodological scope and limitations.

Pharmacology of ketamine

Ketamine’s pharmacological profile is defined by complex interactions across multiple receptor systems, which together explain its anesthetic, analgesic, and neuroprotective properties. Its primary mechanism of action involves non-competitive antagonism at N-methyl-D-aspartate (NMDA) receptors, which play a crucial role in synaptic plasticity and memory function. This NMDA blockade underlies ketamine’s dissociative and anesthetic effects by reducing excitatory neurotransmission and limiting excessive glutamatergic activity [6,7].

In addition to NMDA receptor antagonism, ketamine interacts with the opioid system, a mechanism believed to contribute to its potent analgesic effects. Evidence suggests a synergistic relationship between NMDA and opioid receptors, enhancing ketamine’s efficacy in pain management, particularly in acute and procedural contexts [6].

Ketamine also modulates monoaminergic and cholinergic systems. Monoaminergic pathway modulation is associated with its antidepressant effects, while activity on cholinergic circuits contributes to broader psychotropic and cognitive effects [8]. Together, these interactions produce ketamine’s characteristic dissociative state and support its neuroprotective capacity by modulating excitatory neurotransmission and preventing glutamate-induced neurotoxicity [9].

Pharmacokinetically, ketamine is rapidly absorbed and widely distributed throughout the body. Its high lipid solubility enables efficient crossing of the blood-brain barrier, facilitating central nervous system activity [10]. Hepatic metabolism occurs primarily via cytochrome P450 enzymes CYP3A4 and CYP2B6, generating active metabolites such as (2R,6R)-hydroxynorketamine. These metabolites retain some therapeutic effects of the parent compound but exhibit fewer dissociative properties [10,11]. Ketamine can be administered IV, intramuscularly (IM), intranasally (IN), or orally, with IV administration producing the fastest onset. Its pharmacodynamic profile features a rapid onset and short duration of effect, with a clinical half-life of approximately two to three hours depending on the route [7,8].

Compared with other sedatives commonly used in emergency medicine, such as midazolam, propofol, and etomidate, ketamine offers several advantages. It preserves airway reflexes and provides intrinsic analgesia, making it particularly suitable for trauma and emergency procedural sedation [9]. Its rapid onset and combined sedative-analgesic profile enhance procedural efficiency and patient comfort. However, ketamine’s dissociative effects and potential for psychological disturbances or abuse require careful clinical consideration [11].

Clinical indications in the ED

During fracture and dislocation reductions, ketamine provides effective analgesia and sedation, enabling smooth realignment of bone structures with minimal patient distress. This balance of analgesia and sedation is particularly valuable in high-stress environments such as emergency and trauma care, where rapid, safe, and efficient procedures are essential.

In complex wound repair, especially among pediatric populations, ketamine has demonstrated high efficacy even without local anesthetics. Studies indicate that ketamine sedation alone can provide sufficient analgesia for laceration repairs, minimizing procedural pain while maintaining patient cooperation [12]. By inducing a dissociative state rather than complete unconsciousness, ketamine ensures patient comfort and procedural control. Among children, it remains a preferred sedative due to its strong safety profile and predictable pharmacodynamics. Comparative analyses have shown it to be non-inferior to opioids such as morphine and fentanyl for pediatric pain management, with transient side effects that rarely require clinical intervention [13].

Beyond procedural sedation, ketamine is widely used in acute pain and trauma management. In cases of major trauma, burns, or polytrauma, it provides rapid and effective analgesia while maintaining hemodynamic stability. Evidence suggests ketamine is associated with a lower incidence of adverse events compared with opioids, particularly in prehospital or resource-limited settings where rapid pain control is critical [14]. Its sympathomimetic properties are especially advantageous in patients with hypotension or shock, as ketamine maintains cardiovascular tone and supports perfusion without causing significant respiratory depression. At low doses, ketamine offers effective analgesia with minimal hemodynamic compromise, representing a safer alternative to opioids in hemodynamically unstable patients [15,16].

Ketamine also plays a key role in airway management, particularly during RSI. Its ability to preserve airway reflexes while providing sedation and analgesia distinguishes it from other induction agents. During pre-intubation, ketamine facilitates patient cooperation and muscle relaxation without the marked respiratory depression observed with benzodiazepines or propofol [17]. Furthermore, its favorable hemodynamic profile reduces the risk of hypotension during intubation, offering an advantage over agents that commonly cause vasodilation or myocardial depression [18].

Age-specific considerations are essential when using ketamine. In pediatric patients, dosing must be carefully calculated based on age and weight to minimize adverse events while ensuring adequate sedation and analgesia. In geriatric populations, ketamine’s dissociative properties and minimal respiratory suppression make it a safer alternative to traditional sedatives; however, dose adjustments and careful monitoring are necessary to account for age-related pharmacokinetic and pharmacodynamic changes that may affect drug distribution and clearance [14].

Adverse effects and safety profile

Ketamine’s cardiovascular and respiratory effects are key components of its clinical profile, offering advantages in emergency medicine while requiring careful monitoring for potential complications. One of its most notable benefits is the preservation of airway reflexes, which distinguishes it from many other sedative agents. This property makes ketamine particularly valuable for emergency sedation, where maintaining spontaneous ventilation and airway protection is critical. However, despite this advantage, airway complications such as laryngospasm can occur, especially in vulnerable populations such as children or patients with airway hyperreactivity [18].

In addition to its respiratory profile, ketamine exerts significant cardiovascular effects due to its sympathomimetic activity. By increasing catecholamine release, it can elevate heart rate and blood pressure, producing tachycardia and hypertension in some patients. These effects are generally well tolerated in healthy individuals and can even be advantageous in hypotensive or shock states. Nevertheless, they may pose risks in patients with pre-existing cardiovascular disease, where increased myocardial workload could exacerbate ischemia or trigger arrhythmias [19,20].

Beyond physiological effects, ketamine is associated with a distinct spectrum of psychological and emergence reactions. Its dissociative mechanism, while therapeutically useful, may cause agitation, hallucinations, or vivid dreams during recovery. Although often transient, these reactions can lead to patient discomfort or distress [2,4]. Coadministration of benzodiazepines can mitigate these effects, providing anxiolysis and sedation. This strategy reduces both the frequency and intensity of emergence phenomena and enhances overall patient comfort and recovery quality [20].

Several contraindications should be considered when using ketamine for sedation or analgesia. Its sympathomimetic properties make it less suitable for patients with cardiovascular disease or elevated intracranial or intraocular pressure, as these conditions may be exacerbated by the drug’s hemodynamic effects [21]. Ketamine should also be avoided in individuals with active psychosis or stimulant intoxication, as it can worsen psychiatric symptoms and provoke unpredictable behavioral responses [17].

Dosing and administration protocols

Ketamine dosing and administration in emergency medicine vary according to the clinical objective, route of delivery, and patient characteristics. IV administration is commonly employed for analgesia at sub-dissociative doses of 0.15-0.30 mg/kg, both of which demonstrate comparable efficacy and similar adverse effect profiles [22]. This route allows rapid onset and precise titration, making it ideal for procedural sedation and short-term pain control.

IM administration is frequently used for acute agitation, with effective sedation achieved at doses ranging from 2 to 5 mg/kg. Both lower (~2 mg/kg) and standard (4-5 mg/kg) doses provide adequate sedation, offering flexibility depending on the patient’s level of agitation and clinical stability [4]. The IN route offers an alternative for pain management, yielding analgesic effects comparable to IV opioids, although specific dosing parameters are less well established in the literature [23].

Continuous physiological monitoring is essential during ketamine administration, as even sub-dissociative doses may cause adverse reactions such as respiratory depression, agitation, or hemodynamic fluctuations [4,20]. Vital signs, oxygen saturation, and cardiac rhythm should be closely observed throughout the procedure and recovery period. Airway management equipment, including oxygen delivery systems, suction, and intubation tools, must be immediately accessible, particularly when higher doses are administered or when patients present with compromised respiratory function [24].

Titration strategies are often necessary, as initial dosing may not achieve the desired level of sedation or analgesia. Incremental dosing allows clinicians to individualize treatment according to patient response, minimizing the risk of oversedation or adverse events. In some cases, adjunctive sedatives such as benzodiazepines are coadministered to enhance sedation and reduce the incidence of emergence reactions or agitation during recovery [20].

Institutional protocols and international guidelines consistently endorse ketamine as an appropriate agent for emergency sedation. The American College of Emergency Physicians recognizes its utility for rapid sedation in severely agitated patients, emphasizing adherence to appropriate dosing ranges and continuous monitoring to ensure safety [20]. Similarly, the National Institute for Health and Care Excellence and the World Health Organization support ketamine’s inclusion in emergency care protocols, particularly in contexts requiring rapid, effective sedation where airway preservation and hemodynamic stability are priorities [4].

Clinical evidence and comparative studies

The efficacy and safety of ketamine in emergency medicine have been extensively evaluated, particularly in comparison with other sedative agents such as etomidate, midazolam, and propofol. During RSI, ketamine has been associated with slightly greater hemodynamic variability compared to etomidate; however, it offers important clinical advantages, including a reduced need for vasopressors and a lower risk of adrenal suppression [25]. In critically ill patients, ketamine demonstrates a moderate probability of reducing mortality compared with etomidate, although secondary outcomes, such as organ failure scores and intubation success rates, do not differ significantly between the two agents [26].

When compared with benzodiazepine-opioid combinations, ketamine demonstrates a more favorable respiratory safety profile, producing fewer respiratory adverse events. This advantage is clinically relevant in emergency settings, where the preservation of spontaneous ventilation is critical. Nonetheless, when combined with propofol, ketamine may increase the incidence of transient neurological side effects, highlighting the importance of careful titration and monitoring in combination regimens [27].

Recovery time and patient satisfaction are additional factors influencing the choice of sedative in emergency care. Propofol alone is associated with the shortest recovery time among commonly used agents, reflecting its rapid offset of action. However, combinations of ketamine and propofol, often referred to as “ketofol,” are associated with higher patient satisfaction scores, likely due to improved analgesia and reduced procedural discomfort [27].

Comparative studies between ketamine and etomidate in specific patient populations, such as those with sepsis, have shown no significant differences in overall survival rates. However, etomidate use is linked to higher early vasopressor requirements, reflecting its potential to induce adrenal suppression [28]. In contrast, ketamine’s cardiovascular profile, characterized by preserved sympathetic tone and maintained perfusion, appears more favorable in critically ill or hypotensive patients, potentially contributing to reduced mortality in those requiring intubation under unstable conditions [27].

## Conclusions

Ketamine represents a highly effective and safe option for procedural sedation and analgesia in emergency medicine, combining rapid onset of action, preservation of airway reflexes, and hemodynamic stability, making it particularly valuable in trauma and critically ill patients. Its multifaceted pharmacological profile, characterized by NMDA receptor antagonism and modulation of opioid, monoaminergic, and cholinergic systems, explains its unique balance of sedation, analgesia, and neuroprotection, distinguishing it from traditional sedatives. Despite its benefits, ketamine use requires careful patient selection, adherence to standardized dosing protocols, and continuous monitoring to prevent adverse psychological or cardiovascular effects. When properly managed, it remains an indispensable agent for safe and effective procedural sedation in the ED.
